# Prevalence and diagnostics of congenital malaria in rural Burundi, a cross-sectional study

**DOI:** 10.1186/s12936-016-1478-0

**Published:** 2016-08-30

**Authors:** Jorgen Stassijns, Wilma van den Boogaard, Pieter Pannus, Alphonse Nkunzimana, Anna Rosanas-Urgell

**Affiliations:** 1 Brussels Operational Centre, Médecins sans Frontières, 46 Rue de l’Arbre Bénit, 1050 Brussels, Belgium; 2 Operational Research Unit, Brussels Operational Centre, Médecins sans Frontières, Luxembourg City, Luxembourg; 3 Brussels Operational Centre, Médecins sans Frontières, Kirundo, Kirundo Province Burundi; 4 Institute of Tropical Medicine, Antwerp, Belgium; 5 Ministry of Health, Province Sanitaire de Kirundo, Kirundo, Kirundo Province Burundi

**Keywords:** Congenital malaria, Prevalence, Diagnosis, Rapid diagnostic test, Burundi

## Abstract

**Background:**

Congenital malaria, defined as the presence of asexual forms of malaria parasites in the peripheral blood during the first 7 days of life, remains a neglected area of research. Knowledge gaps exist about prevalence and management of malaria in this age group. The objective of this study was to evaluate the prevalence of congenital malaria and the validity of a rapid diagnostic test (RDT) for its diagnosis in rural Burundi.

**Methods:**

A cross-sectional study was conducted in a meso-endemic malaria context in Burundi among 290 mothers, and their newborns (n = 303), who delivered at the maternity departments of Kirundo and Mukenke Hospitals during March and April 2014. Peripheral blood samples were collected from all mothers/newborns pairs in order to examine the presence of malaria parasites with two RDT (SD-Bioline HRP2 and Carestart pan-pLDH) and a blood slide. In addition, quantitative real-time polymerase chain reaction (PCR) was performed from the newborn peripheral sample. Frequencies and proportions were calculated for categorical variables. Sensitivity and specificity were calculated with a 95 % confidence interval (CI).

**Results:**

None of the newborns were found positive by PCR (0/303; 95 % CI 0.0–1.3). The prevalence in newborns born from microscopy-positive mothers was 0 % (0/44; 95 % CI 0.0–8.0). Two newborns were positive with SD-Bioline HRP2 (0.7 %, 95 % CI 0.2–2.4) but none with Carestart pan-pLDH or microscopy. Sensitivity of the diagnostic tests could not be evaluated as no congenital malaria was detected. Specificity of SD-Bioline HRP2, Carestart pan-pLDH and microscopy to detect congenital malaria was 99.3 % (95 % CI 97.6–99.8), 100.0 % (95 % CI 98.3–100.0) and 100.0 % (95 % CI 98.8–100.0), respectively.

**Conclusion:**

In Burundi or the Central African region, no recent prevalence studies for congenital malaria have been carried out. This study found that the prevalence of congenital malaria in two hospitals in Kirundo province is zero. RDT showed to have an excellent specificity and, therefore, can be used to rule out congenital malaria: the risk of overtreatment is low. However, as no cases of congenital malaria were detected, the study was not able to draw conclusions about the sensitivity of the RDT, nor about risk factors for congenital malaria. Further studies evaluating the sensitivity of RDT for diagnosis of congenital malaria are needed.

## Background

Congenital malaria is defined as the presence of asexual parasites in the cord blood or peripheral blood during the first week of life, due to the transmission of parasites through the placenta just before or during the delivery [1]. It remains a neglected area of research with knowledge gaps concerning the prevalence, the clinical presentation, the risk factors, the diagnosis or treatment [2].

The prevalence of congenital malaria in sub-Saharan Africa is estimated between 0 and 54 % [3, 4]: the wide range could be attributed to differences in the definition of congenital malaria, the endemicity of the region and the level of maternal immunity, the methods and characteristics of the diagnostic tool used (microscopy or polymerase chain reaction) or the type of specimen collected (cord blood or peripheral blood) [2].

Because of the effectiveness of the placenta as a barrier, the presence of maternal antibodies and the protective effect of foetal haemoglobin (Hb F), congenital malaria has long been considered as a rare event [2]. Its true burden however might be underestimated because of the non-specific clinical picture and the absence or the delayed presentation of symptoms. It is reported that it might take 3–4 weeks before congenitally-infected infants present symptoms [5] and only 34 % of parasitaemic newborns would present symptoms within 3 days [6]. Other reasons for underestimating the burden of congenital malaria are the lack of awareness among clinicians, overall underreporting or the lack of adequate diagnostic tools. PCR is more sensitive than a blood smear for the detection of congenital malaria, however it is not clear whether a positive PCR-result indicates active infection [7].

Neonates usually present with a low to very low parasitaemia: a study carried-out in four meso- and hyper-endemic regions in Nigeria found a mean parasite density of 48 parasites/µL (range: 8–200), with 62 % of the neonates spontaneously clearing infection within 2 days [6]. In Accra, Ghana newborns presented with ≤50 parasites/µL [8] and in Burkina Faso, the mean parasite density among newborns was 316 parasites/µL [9].

Microscopy is the main diagnostic tool currently used for the diagnosis of congenital malaria but access to quality microscopy is often problematic in low-income contexts, even in hospital settings. PCR is considered as the most sensitive tool but is mainly used for research purposes. Rapid diagnostic tests (RDT) are the most widely used diagnostic tests for malaria; they are easy to use, cheap and reliable and can, therefore, be used at peripheral levels. However evidence is lacking about the use and validity of RDT for the diagnosis of congenital malaria; to our knowledge only one study compared the use of PCR, microscopy and RDT (HRP2/pLDH and First Response Combo) [8]. No cases of congenital malaria were detected by microscopy and RDT among 522 newborns, but 12 % were found positive by PCR. No study has reported the sensitivity, specificity or predictive values of RDT in neonates.

In Burundi, a sub-Saharan African country known to have a very high malaria burden [10], Médecins Sans Frontières (MSF) treated in 2013 3476 cases of severe malaria in a Ministry of Health (MSPLS) supported hospital in the North-Eastern province of Kirundo. The health centres treated over a half million cases of simple malaria using RDT for diagnostics and artemisinin-based combination therapy (ACT) as first line-treatment. Country-wide 8023 cases of malaria in pregnancy were reported, and congenital malaria had never been diagnosed [10].

The main objectives of our study were to define the prevalence of congenital malaria among newborns in Kirundo Province, Burundi, and to assess the validity of RDT for the diagnosis of congenital malaria.

## Methods

### Study population

All mothers with their newborns who were born in Kirundo and Mukenke district hospitals between March and April 2014.

### Study settings

Burundi is a Central African country with a population of approximately 10.5 million people. The Northern part is considered as a meso-endemic zone for malaria with transmission throughout the year and peaks during the two rainy seasons (February–May and October–December). Since 2013 MSF provides support to the MSPLS in the Province Sanitaire de Kirundo, with a main focus on severe malaria.

### Study design

This was an observational cross-sectional study carried out in the maternity and paediatric services of Kirundo and Mukenke district hospitals in Kirundo-Province, Burundi.

### Sample size and inclusion/exclusion criteria

The prevalence of malaria among children 6–59 months is estimated at 30.6 % using RDT (16.3 % *Plasmodium falciparum;* 14.3 *% P. falciparum* and/or mixed infections and 0 % non-*P. falciparum*) and at 23.8 % using microscopy (20.7 % *P. falciparum*; 2.4 % mixed infections and 0.7 % non-*P. falciparum*) [11]. Data concerning the prevalence of congenital malaria in Burundi are not available. For the RDT-validity study, in order to detect *P. falciparum* with a sensitivity of 90 %, 138 PCR(+) samples were needed (α error 0.05, precision 5 %). Similar calculations applied for the specificity: a number of 138 PCR(−) samples were required.

Inclusion criteria were: 1/mothers and their newborns of 0–7 days, born in the maternity-department 2/children 0–7 days old admitted in the paediatric department and their mothers. Excluded were children of 0–7 days having received anti-malaria treatment since birth.

### Blood sample collection procedures

Approximately 100 µL of capillary blood was collected from mothers and newborns through finger-prick or heel-prick, within 18 h after delivery, for testing with RDT, thick smear/thin smear examination and PCR.

The performance of two different types of RDT was evaluated: a histidine rich protein (HRP2)-based test and a parasite lactate dehydrogenase (pLDH)-based test. The HRP2-test was the SD Bioline Malaria Antigen Pf (HRP2) (Manufacturer: Standard Diagnostics Inc.), currently used in Burundi as well as in many other contexts. The pLDH-test evaluated was the Carestart Malaria pLDH (pan) (Manufacturer: Access Bio Inc.). Quantitative PCR (qPCR) was used as gold standard.

Thick and thin smears were prepared at the bedside from two small drops of blood applied on the same glass slide directly from the finger- or heel prick. Each thick smear was analysed with light microscopy during 6 min to determine parasitaemia. For positive thick smears, the corresponding thin smear was analysed to determine the Plasmodium species. All smears were analysed by two readers. In case of a discordant result, a third reader analysed the slide to obtain a final result. An expert microscopist reread 10 % of the samples as a quality control.

For the molecular diagnosis (qPCR), filter papers with dried blood samples were punched, and one circle of 5 mm in diameter was used for DNA extraction with a QIAamp DNA blood extraction kit (Qiagen) following the manufacturer’s recommendations. Extracted DNA was eluted in 150 µL water and used for quantitative real time PCR (qPCR). *Plasmodium falciparum* qPCR targeting 18S gene was performed as described elsewhere [12]. The lower limit of detection using filter paper extracted DNA was 10 parasites/µL.

### Questionnaire

Data were collected about the clinical condition and the outcome of the newborns, the clinical condition of the mothers, the history of malaria during the last trimester and the use of long-lasting insecticide-treated nets (LLIN) during pregnancy.

### Informed consent, confidentiality and data analysis

Individual consent was obtained from the mother or in absence of the mother the representative of the eligible children. Separate information sheets and consent forms were used for the prevalence and the validity study and were piloted beforehand to ensure they were easy to understand by the patients. The information sheet was read and explained to each eligible mother or to the representative of each eligible child. Informed consent was in writing, through signature on the consent declaration or, in case the study participant or legal representative was illiterate, through a mark. The study participant or representative was given a copy of the information sheet and consent form.

All participant records were given an identification code. Only the anonymous codes were entered in a dedicated Microsoft Excel (version 2010) database, thus participants could not be identified. Patient records and dedicated electronic database were only accessible to the principal investigator and data encoder. All encoded data related to the study objectives were crosschecked for validation with the questionnaires and laboratory registers. Data analysis were performed by using Epidata analysis software (version 2.2.2.182, EpiData Association). Baseline characteristics were described using medians and interquartile ranges (IQRs) for continuous variables and counts and percentages for categorical data. Sensitivity, specificity, positive predictive value (PPV) and negative predictive value (NPV) variables were calculated with a 95 % confidence interval.

## Results

During the study period a total of 290 mothers delivered in Kirundo and Mukenke maternity wards of a total of 303 newborns. All 290 mothers were willing to participate, thus 290 mothers and 303 newborns were included in the study. The characteristics of the study participants are summarized in Table 1. Among the mothers 25 % reported a malaria episode during the last trimester of pregnancy of which all of them had been treated. Only 26 % slept under a LLIN the night before admission in the hospital. While 44 (15 %) were tested microscopy-positive for *P. falciparum* with a mean parasitaemia of 483 parasites/µL (ranges: 64–490667) during their delivery, only 6 % had a fever (>38.2 °C). Among the newborns, 85 (28 %) had a low birthweight (<2500 g) and 2 % were observed with a fever. No newborns suspected with congenital malaria were admitted in the paediatric service during the study.

**Table 1:** Demographic and clinical characteristics of mothers and newborns, Kirundo and Mukenke hospitals, Burundi (March–April 2014)

Characteristics	Mothers	Newborns
	N = 290 (%)	N = 303 (%)
Age (median; IQR)	25 years (21–30)	1 day (1–1)
Gender		
Male	–	148 (50)
Female	–	150 (50)
Birthweight in grams (median; IQR)	–	2900 (2200–3200)
Gravidity (median; IQR)	3 (1–5)	–
Clinical signs	N = 276 (%)	N = 292 (%)
Fever	15 (6)	5 (2)
Hypothermia	–	33 (11)
Severe anaemia	6 (3)	ND
Outcomes		
Malaria in pregnancy	109 (38)	–
Malaria 3rd trimester	73 (25)	–
Malaria treated (n = 73)	73 (100)	–
Possession LLIN	179 (62)	–
Slept under LN night before admission	76 (26)	–

*IQR* interquartile range, *ND* no data, *LLIN* long-lasting insecticide-treated nets

None of the newborns were found positive by PCR (0/303, 95 % CI 0.0–1.3) (Table 2). The prevalence in newborns from microscopy-positive mothers was 0 % (0/44, 95 % CI 0.0–8.0). Two newborns were positive with the SD-Bioline HRP2 (0.7 %, 95 % CI 0.2–2.4) while none of the newborns were found positive with the Carestart pan-pLDH or microscopy. Both newborns were asymptomatic, received treatment and were discharged. The RDT positive neonates were from two mothers with a high parasitaemia (99,520 and 454,061 parasites/µL respectively) and positive SD Bioline HRP2 and Carestart pan-pLDH results. Both mothers had no fever or other symptoms, but reported to have had malaria during the 3rd trimester of pregnancy which was treated with ACT. Because of supply problems during the study a proportion of newborns and mothers was not tested with the Carestart pan-pLDH RDT.

**Table 2:** Results diagnostic tests in mothers and newborns, Kirundo and Mukenke district hospitals, Burundi (March–April 2014)

Mothers	N	Positive N (%)
Rapid diagnostic test		
SD bioline HRPII	286	85 (30)
Carestart pan-pLDH	204	23 (11)
Microscopy	285	44 (15)

Newborns	N	Positive N (%)
Rapid diagnostic test		
SD bioline HRPII	294	2 (0.7)
Carestart pan-pLDH	210	0
Microscopy	296	0
RT-PCR	303	0

*SD* standard diagnostics, *HRPII* histidine rich protein, *pLDH* parasite lactate dehydrogenase, *RT-PCR* real-time polymerase chain reaction

As no congenital malaria was detected with qPCR it was not possible to evaluate the sensitivity of the diagnostic tests. However, the specificity of the SD-Bioline HRP2, the Carestart pan-pLDH and microscopy to detect congenital malaria was 99.3 % (95 % CI 97.6–99.8), 100.0 % (95 % CI 98.3–100.0) and 100.0 % (95 % CI 98.8–100.0), respectively (Table 3). Because of the absence of qPCR positive newborns it was decided not to perform qPCR on the samples of the mothers.

**Table 3:** Performance RDT and microscopy in mothers and newborns relative to qPCR as gold standard

	SD bioline HRPII	Carestart pan-pLDH	Smear
Sensitivity	–	–	–
Specificity	99.3 % (95 % CI 97.6–99.8)	100 % (95 % CI 98.3–100)	100 % 95 % CI 98.8–100)
PPV	0.0 % (95 CI 0.0–84.2)	–	–
NPV	100 % (95 % CI 98.7–100)	100 % (95 % CI 98.3–100)	100 % (95 % CI 98.8–100)

Kirundo and Mukenke hospitals, Burundi (March–April 2014)

*PPV* positive predicted value, *NPV* negative predicted value, *CI* confidence interval, *SD* standard diagnostics, *HRPII* histidine rich protein, *pLDH* parasite lactate dehydrogenase

## Discussion

The results of this study show a zero prevalence of congenital malaria among newborns in two hospitals in the meso-endemic region of Kirundo. Due to the absence of qPCR-positive cases, the sensitivity of the RDT and the risk factors for congenital malaria could not be assessed. Both types of RDT and microscopy had a very high specificity and can therefore be used to rule out congenital malaria; the risk of giving unnecessary treatment is low.

Only one other study used RDT for the diagnosis of congenital malaria, in which none of the newborns was found RDT-positive. Nothing was reported on RDT sensitivity/specificity [8].

The prevalence of congenital malaria found in our study may be explained by several factors. First, a zero prevalence seems surprising because the survey was carried-out during the wet, high transmission season in a meso-endemic malaria setting. On the other hand it might be assumed that mothers had acquired a higher level of immunity during the high transmission season and their malaria specific antibody responses were boosted when exposed to malaria infection. The transplacental passage of these maternal antibodies could have contributed to the clearance of congenital infections occurring among newborns of high-transmission settings. However there seems to be no consensus about the effect of the level of malaria endemicity or the malaria season on the prevalence of congenital malaria, as some studies report a higher prevalence during the wet season [13, 14], while others suggest that newborns are more protected during the transmission season [4, 15].

Secondly, none of the newborns born from RDT or microscopy-positive mothers was found to be qPCR-positive, not even from mothers with very high parasitaemia, which could confirm the role of the placenta as effective barrier. The fact that two newborns from mothers presenting with high parasitaemia tested positive with the HRP-2 RDT, but both qPCR-negative, suggests that the HRP-2 antigen could pass the placenta, causing the RDT to be positive, while the deoxyribonucleic acid (DNA) is not transmitted. This study could not confirm that newborns from mothers with high parasitaemia are more likely to have parasites [16].

Third, the measures to prevent malaria in pregnancy are poorly implemented. A nationwide survey carried-out in 2012 reported that only 52 % of the pregnant women in the Northern Region slept under a LN the night before the survey [11]. The use of Antenatal Care (ANC) is low: in 2013 the coverage of ANC3 was 60 % while 35 % of women had their first ANC-visit only in their third trimester of pregnancy [10]. The Intermittent Preventive Treatment in pregnancy (IPT-p), recommended by the World Health Organization (WHO), was not yet implemented in Burundi in 2013. A combination of high transmission and low coverage of preventive measures results in high exposure to malaria infection and possibly a considerable prevalence of congenital malaria. The reasons why the prevalence of congenital malaria was found so low in Kirundo region, despite the high transmission and low coverage of preventive interventions, are not fully understood.

Since the year 2000, studies on congenital malaria have been carried-out mainly in western Africa and recent data about the occurrence of congenital malaria in the Central African region are not available. The prevalence found in this study is similar to the low prevalence of 1.4 % reported in Burkina Faso [9] or 2.2 % reported in Ghana [8], but lower than the numbers reported from studies in Nigeria [6, 17–20]. However comparisons between studies are difficult to make due to the different methodologies being used (type of sample or type of diagnostic test), the different study populations being observed (symptomatic versus asymptomatic newborns) or the different contextual levels of malaria endemicity. Moreover, a review of the studies reporting the highest rates showed gaps in reporting or conduct of the laboratory procedures [19].

This study also had some limitations. First, it was not feasible to collect samples from placental and cord blood. Other studies reported various results depending on the type of sample collected, and in studies collecting both cord blood and peripheral blood the frequency or level of parasitaemia was lower in peripheral blood than in cord blood [2, 4, 19, 21–23]. Thus the collection of peripheral blood may have underestimated the prevalence of congenital malaria in the study population.

Secondly, mothers in Burundi leave the hospital within a maximum of 24 h after birth, often between 6 and 12 h, therefore, only one sample could be taken at birth. Several authors recommend repeated sampling to evaluate the presence of congenital malaria; the prevalence was found lower in samples collected within a few hours after birth, compared to sampling on consecutive days [18, 19]. Others recommend not to exclude malaria until three negative smears have been obtained over 48 h [23, 24].

Third, no sick newborns suspected of congenital malaria were admitted in the paediatric ward during the whole study period, and thus the prevalence of congenital malaria among symptomatic newborns could not be evaluated.

Fourth, this study only assessed the prevalence among newborns born in Kirundo and Mukenke Hospitals. From all expected deliveries, about one out of four still take place at home [10] and, therefore, the study cannot account for the prevalence of congenital malaria among those.

Finally, the fact that the same laboratory technicians performed the RDT as well as the microscopy analysis might have introduced a certain bias.

## Conclusions

This is the first study which has evaluated the prevalence of congenital malaria in Burundi, and no studies have been carried-out recently in the Central African region. The prevalence of congenital malaria was found to be zero. It was also one of the first studies evaluating the performance of RDT for the diagnosis of congenital malaria. As their specificity was very high, RDT can be used to rule out congenital malaria; the risk of giving unnecessary treatment is low. However further research is needed for evaluating the sensitivity of the RDT or the risk factors for congenital malaria.

## Abbreviations

ACT: artemisinin-based combination therapy
ANC: antenatal care
HRP2: histidine-rich protein
IPT-p: intermittent preventive treatment in pregnancy
IQRs: interquartile ranges
LLIN: long-lasting insecticide-treated nets
MSF: Médecins sans Frontières
MSPLS: Ministère de la Santé Publique et de la Lutte contre le Sida/Ministry of Health
NPV: negative predictive value
pLDH: parasite lactate dehydrogenase
PCR: polymerase chain reaction
PPV: positive predictive value
qPCR: quantitative real time PCR
RDT: rapid diagnostic test
SD: standard diagnostics
